# Age and liver transplantation: a key factor in clinical outcomes? single center study in Argentina

**DOI:** 10.3389/fragi.2025.1729048

**Published:** 2025-12-11

**Authors:** Marco Santillán Pazmiño, Paula Constanza Arriola Benitez, Maria Florencia Fernandez, Silvina Yantorno, Valeria Descalzi, Franco Daniel Tarditti, María Lucia Novellis, Juan Cruz Fasolo, Martín Rumbo, Stefan Günther Tullius, Pablo Barros Schelotto, Gabriel Eduardo Gondolesi, María Virginia Gentilini

**Affiliations:** 1 Instituto de Medicina Traslacional, Trasplante y Bioingeniería (IMeTTyB- CONICET- Universidad Favaloro), Buenos Aires, Argentina; 2 Cancer Immunobiology Laboratory, Facultad de Ciencias Biomédicas, Instituto de Investigaciones en Medicina Traslacional (IIMT), CONICET-Universidad Austral, Buenos Aires, Argentina; 3 Cirugía General, Hepatología y Trasplante Hepático, Hospital Universitario Fundación Favaloro, Buenos Aires, Argentina; 4 Instituto de Estudios Inmunológicos y Fisiopatológicos, IIFP, Universidad Nacional de La Plata, CONICET, La Plata, Argentina; 5 Division of Transplant Surgery, Department of Surgery, Brigham and Women’s Hospital, Harvard Medical School, Boston, MA, United States; 6 Medstar Georgetown Transplantation Institute, Georgetown University Hospital, Washington, DC, United States

**Keywords:** liver transplant, aging, senesence, organ allocation, elderly donor, younger donor

## Abstract

**Introduction:**

Growing demand for liver transplantation (LTx) has increased the use of elderly donors. In Argentina, however, data on the clinical impact of donor age on post-transplant outcomes remain limited.

**Objective:**

To evaluate the effect of donor age on clinical, functional, and molecular outcomes after LTx at Hospital Universitario Fundación Favaloro Buenos Aires, Argentina.

**Methods:**

We performed a retrospective cohort study of 494 LTx conducted between 2009 and 2020. Patients were stratified into two age groups: 18–59 years (Younger) and ≥60 years (Elderly). Overall and graft survival (OS and GS) were assessed using Kaplan–Meier and Cox regression analyses adjusted for recipient age, donor age, recipient gender, donor gender, transplant year, MELD score, disease etiology, donor BMI, DRI, CIT, WIT, Total Bilirubin (TBIL) and INR. Early postoperative complications including early allograft dysfunction (EAD) and early renal replacement therapy (RRT) were evaluated. Post-transplant liver function was assessed by routine biochemical tests. Gene expression of pro-inflammatory and senescence markers was quantified by qRT-PCR, and lipofuscin deposition was measured using ImageJ.

**Results:**

After applying exclusion criteria, 267 LTx were included (Younger donors: n = 222; Elderly donors: n = 45). Recipients of elderly donor grafts showed significantly lower OS and GS (p < 0.05). In the multivariable analysis, donor age and TBIL remained independent predictors of GS, whereas donor age, recipient age, and TBIL were associated with OS. In contrast, neither the incidence of EAD nor early RRT differed between recipients of elderly versus young donor grafts. Early postoperative biochemical profiles were also similar between groups, with no significant differences in ALT, AST, ALP, or TBIL levels. Molecular analyses demonstrated that elderly donor livers exhibited significantly higher expressions of IL-1β, IL-6, TNF-α, p21 and CCND1, Elderly donor livers displayed higher baseline lipofuscin accumulation (p < 0.05), consistent with age-associated cellular senescence, and trends toward higher rejection rates.

**Conclusion:**

Donor liver aging, characterized by increased inflammatory and senescence signatures, is associated with reduced patient and graft survival. These findings underscore the clinical relevance of considering donor biological age, beyond chronological age, in organ allocation and selection strategies.

## Introduction

Liver transplantation (LTx) is a life-saving therapeutic intervention for patients with end-stage liver disease and acute liver failure (Kwong et al., 2024; Dutkowski et al., 2015). Advances in surgical techniques, organ preservation methods, immunosuppressive therapies, and perioperative care have substantially improved post-transplant survival rates, rendering LTx a viable treatment option for an increasing number of patients (Tingle et al., 2023; Banker et al., 2023; Vimalesvaran et al., 2024). However, the growing demand for donor organs, exacerbated by an aging global population, the used of donors after cardiovascular death, and the increasing use of regional normothermic perfusion or *ex-vivo* machine perfusions, has necessitated a re-assessment of age-related factors in LTx (van Beekum et al., 2021; Mergental et al., 2020; Boeva et al., 2021; Durand et al., 2008; Garcia-Valdecasas, 2012; van Leeuwen et al., 2019; Reiling et al., 2020).

The influence of donor age on LT outcomes remains a topic of ongoing debate (Busquets et al., 2001; Levy et al., 2001). Although numerous studies have investigated the use of grafts from elderly donors, comprehensive multivariate analyses remain limited, and randomized control trials difficult to do, therefore no definitive age threshold or criteria has been established to determine graft suitability (Houben et al., 2022; Caso-Maestro et al., 2018). The growing number of transplant candidates and the persistent organ shortage have necessitated the incorporation of marginal liver grafts, including those from elderly donors. This shift underscores the need to understand immunosenescence and its impact on graft survival (GS) and patient outcomes (Heinbokel et al., 2014).

Aging alters immune responses, leading to lower acute rejection rates but higher risks of infections, malignancies, and cardiovascular complications, when the age is analyzed for the recipient (McGovern et al., 2023; Gelson et al., 2010). When aging affects the graft itself, it may reduce regenerative capacity and promote inflammatory and senescence-associated changes, which can further compromise transplant success. As the use of marginal grafts becomes more prevalent, careful evaluation and match with the recipient are essential to obtain the best viability and post-transplant function, along with tailored immunosuppressive strategies to optimize outcomes (Iske et al., 2024a; Iske et al., 2024b; Martins et al., 2014).

Given the growing reliance on elderly donors due to the persistent organ shortage and the lack of published data on their use in our country, this study aims to evaluate the impact of donor age on clinical outcomes in LTx recipients at Hospital Universitario Fundación Favaloro, trying to identify factors affecting GS and recipient outcomes, with the goal of improving LTx strategies, especially for an aging population, and enhancing donor-recipient matching, immunosuppressive management, and perioperative care.

## Materials and methods

### Study overview

**FIGURE 1:**
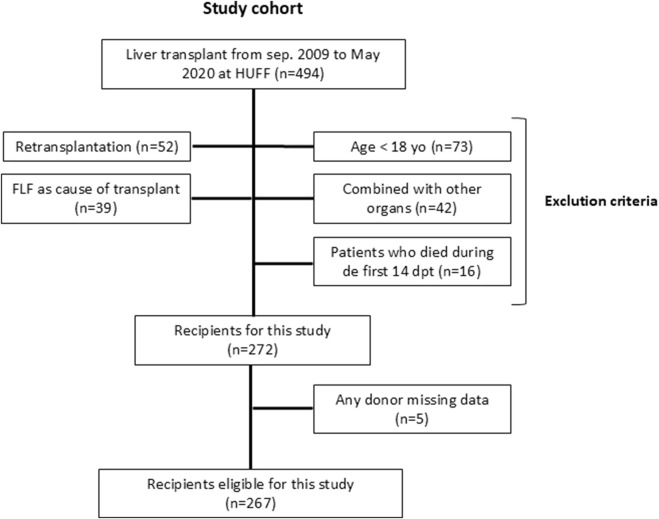
Flow chart of study population selection.

This retrospective single-center study incorporated all liver transplantations performed with donors registered in Hospital Universitario Fundación Favaloro (HUFF), Buenos Aires Argentina, performed from September 2009 to May 2020 (n = 494 liver donors, Figure 1). Exclusion criteria were the following: recipients and donors <18 years old, re-transplants, multivisceral and combined transplant, fulminant hepatic failure, patients who died during the first 14 postoperative days (POD) and missing information about donors. Split liver transplantations were excluded as well as observations with implausible values. Observations with implausible values—defined as physiologically impossible or internally inconsistent entries—were excluded. These included incoherent time intervals, temporally inconsistent dates, demographic values outside eligibility criteria, duplicate records, and extreme or missing laboratory data. All exclusions were confirmed by manual verification to ensure data integrity. Thus, 267 donors-recipients’ pairs were included and divided into 2 groups according to criteria stablished previously (Zetterman et al., 1998) those receiving younger grafts (Younger group, donor 18–59 years old (yo); n = 222) versus those receiving older grafts, considering 60 years the cut-off point (Elderly group, donor ≥60 yo; n = 45). All donors in this cohort were donation-after-brain-death (DBD) donors. Preservation method was uniform (static cold storage), and all grafts were stored in University of Wisconsin (UW) solution. The study protocol was reviewed and approved by the Institutional Ethics Committee, and all procedures were conducted in accordance with the Declaration of Helsinki and relevant national regulations (DDI (1490) 2419; CBE 778719).

### Variables, definitions and data collection

#### Donor and graft variables

For each LTx, the following donor variables were collected: age, gender, height, weight, body mass index, liver graft type, cold ischemia time and cause of death (trauma, anoxia, cerebrovascular accident, other). Additional variables included organ allocation pathway (local, regional, national), donor risk index (DRI), and presence of any reported concerns regarding donor suitability.

The DRI integrates donor and transplant characteristics including donor age, cause of death, donation pathway, graft type, cold ischemia time, and other variables to quantify the relative risk of graft failure. Higher DRI values indicate increased predicted risk. The Model for End-Stage Liver Disease (MELD) score was calculated at the time of transplantation using the standard equation incorporating serum bilirubin, International Normalized Ratio (INR), and serum creatinine, and was used to assess recipient disease severity.

#### Intraoperative and perioperative variables

Variables collected perioperatively included cold ischemia time, post-reperfusion injury, early renal replacement therapy (RRT), and requirement for dialysis during the immediate postoperative period.

Baseline values were defined as the last preoperative measurements. Postoperative values corresponded to routine assessments performed on postoperative day 7 (POD7). All laboratory parameters, including aspartate aminotransferase (AST), Alanine Transaminase (ALT), alkaline phosphatase (ALP), total bilirubin (TBIL), and the International Normalized Ratio (INR) were evaluated at both baseline and POD7.

#### Recipient variables

Recipient characteristics included age at transplantation, gender, etiology of underlying liver disease (categorized autoimmune, cryptogenic cirrhosis, alcoholic liver disease (ALD), metabolic, neoplasia, into viral, other), time post-transplant, MELD at transplant (MELD-Tx), immunosuppressive regimen at discharge and comorbidities. Biochemical variables included ALT, AST, ALP, TBIL and INR.

Early graft outcomes. Early allograft dysfunction (EAD) was defined according to Olthoff et al. (2010). Presence of acute cellular rejection (ACR) within the first-year post-transplant was collected from histopathological reports.

Survival outcomes. Overall survival (OS) and GS times were recorded as follows: date of transplant, year of transplant, date of last follow-up, date of re-transplantation graft failure and cause of death.

All variables were retrieved from institutional electronic health records and verified through manual review of individual clinical charts. Variables with <30% missingness were considered for inclusion in multivariable models.

### Cytokine and senescence markers expression analysis

Liver biopsies were collected at pre-reperfusion time from younger donors (<60 years; n = 9) and elderly donors (≥60 years; n = 5) in RNAlater (Invitrogen) and stored at −80 until processing. Total RNA was isolated using Quick-RNA™ MiniPrep (ZymoReasearch) according to the manufacturer’s instructions. cDNA was obtained using M-MLV Reverse Transcriptase (Invitrogen). Quantitative reverse-transcription polymerase chain reaction (qRT-PCR) analysis was run on a StepOne real-time PCR detection system (Life Technology) using SYBR Green as a fluorescent DNA binding dye. Primer sequences used for amplification were: GAPDH fw 5′-AAG​GTG​AAG​GTC​GGA​GTC​AAC-3′, Rv 5′-GGG​GTC​ATT​GAT​GGC​AAC​AAT​A-3´; TGFB1 Fw 5′-CGA​GCC​TGA​GGC​CGA​CTA​C-3′, Rv 5′-AGA​TTT​CGT​TGT​GGG​TTT​CCA; IL6 Fw 5′-ACT​CAC​CTC​TTC​AGA​ACG​AAT​TG, Rv 5′-CCA​TCT​TTG​GAA​GGT​TCA​GGT​TG-3´; IL10 Fw 5′-AAG​GCG​CAT​GTG​AAC​TCC​C-3′, Rv 5′-ACG​GCC​TTG​CTC​TTG​TTT​TC-3´; IL1B Fw 5′-AGC​TAC​GAA​TCT​CCG​ACC​AC-3′, Rv 5′-CGT​TAT​CCC​ATG​TGT​CGA​AGA​A-3´; TNFα Fw 5′-CGA​GTG​ACA​AGC​CTG​TAG​CC -3′, Rv 5′-GTT​GAC​CTT​GGT​CTG​GTA​GG-3´; p21Waf1/Cip1 Fw 5′-TCA​CTG​TCT​TGT​ACC​CTT​GTG​C-3′, Rv 5′-GGC​GTT​TGG​AGT​GGT​AGA​AA-3´; CCND1 Fw 5′-GCT​GTG​CAT​CTA​CAC​CGA​CA-3′, Rv 5′-TTG​AGC​TTG​TTC​ACC​AGG​AG-3´. Relative transcript levels were calculated using the 2^−ΔCt^ method using as normalizer gene GAPDH.

### Lipofuscin staining and analysis

Lipofuscin, a yellow-brown pigment, often referred to as the “aging pigment”, which accumulates in cells, particularly in those with long lifespans as result of aging was assessed. For the detection of lipofuscin, tissues were retrospectively collected by the pathology department at the pre-reperfusion time, fixed in formalin, and stored in paraffin (Young donor group, n = 6; Elderly donor group, n = 6). Afterwards, they were deparaffinized and rehydrated until reaching distilled water. Subsequently, the slides were incubated in a carbolfuchsin solution for 60 min, after which they were washed with distilled water to remove excess dye. Differentiation was performed by immersing the slides in an acid alcohol solution between 5 and 6 times, until the samples acquire a characteristic pale pink hue. Next, the slides were rinsed under a gentle stream of tap water for 5 min and then washed again with distilled water. For contrast, the slides were immersed in picric acid for approximately 1 min. Following this, the sections were dehydrated through an ascending series of alcohol followed by two consecutive immersions in xylene. The quantification of lipofuscin-positive cells was performed by analyzing 5 randomly selected tissue quadrants. Both lipofuscin-positive and negative cells were counted. Subsequently, the proportion of positive cells relative to the total was calculated, and an average was obtained.

### Statistical analysis

The Kaplan–Meier curve and the Long-rank test were performed to evaluate the predictive capacity of the donor age for liver transplant patient and GS. To analyze the GS probability, when re-transplantation data was required, this date was used to calculate the time until graft failure. When death was given without prior re-transplant, we used the timepoint of death as time until graft lost. If the last observation was a routine follow-up and no re-transplant or death occurred, we considered the patient/graft as censored and calculated the censoring time based on the data of the last follow-up. Comparisons between donor-age groups (young vs. elderly) were performed using χ^2^ or Fisher’s exact test. Unadjusted odds ratios (OR) with 95% confidence intervals were computed. A multivariable logistic regression model for EAD was fitted including donor age (Old = 2 vs. Young = 1) and covariates with <30% missingness and sufficient variability (cold ischemia time, donor gender, MELD score, and perioperative transfusions). Results are presented as adjusted odds ratios with 95% confidence intervals*.* Univariate and multivariate Cox regression analyses were used to examine associations between variables and OS and GS. Statistical analyses and chart creation were done using R 4.1.3 (https://www.r-project.org/) and GraphPad Prism 8.0. Categorical variables were tested for statistical significance, defined as *p* less than 0.05, with the Fisher´s exact test. Normally distributed data were evaluated by a one-way ANOVA with Dunnett’s Multiple Comparison post-test or with an unpaired t-test with Welch’s correction. Non-normally distributed data were evaluated by either a Mann-Whitney test (for pairwise analysis) or by a Kruskal–Wallis test with Dunn’s Multiple Comparison post-test. *p* value designations are as follows: * <0.05, **<0.01, ***<0.001, **** <0.0001.

## Results

### Demographics and clinical characteristics

During the study period, a total of 494 liver transplantations were performed. After applying exclusion criteria, including donors or recipients under 18 years of age (n = 73), re-transplantations (n = 52), multivisceral or combined procedures (n = 42), fulminant hepatic failure (n = 39), early postoperative mortality within 14 days (n = 16), and incomplete donor data (n = 5), a final cohort of 267 donor–recipient pairs was included (Figure 1). Among donors, 222 (83%) were classified as younger donor (18–59 years) and 45 (17%) as elderly donors (≥60 years). The younger donor group consisted of 80 females (36.04%) and 142 males (63.96%), whereas the elderly donor group included 21 females (46.67%) and 24 males (53.33%). All donors tested negative for hepatitis B and hepatitis C.

The demographic and clinical characteristics of the recipients are presented in Table 1. Recipient age differed significantly between groups, with younger recipients averaging 48.18 ± 0.77 years and elderly recipients 65.35 ± 0.29 years. In both groups, most recipients were male (62.5% and 70.43%, respectively), with no significant difference compared to females (*p* > 0.05).

**TABLE 1:** Clinical characteristics of recipient patients.

Characteristic group recipient	Younger N = 152/57% (18–59 years)	Elderly N = 115/43% (>60 years)	p-value
a) Age at time of tx (years)	48.18 ± 0.77	65.35 ± 0.29	0 < 000.1
b) Gender (N)			
Female	57 (37.5%)	34 (29.57%)	0.1937
Male	95 (62.5%)	81 (70.43%)	0.1937
c) Etiology/pathology (N)			
Autoimmune	32 (21.05%)	6 (5.22%)	0.0002
Cryptogenic cirrhosis (unknown etiology)	8 (5.26%)	11 (9.57%)	0.2298
Alcoholic liver disease - ALD	39 (25.66%)	41 (35.65%)	0.0814
Metabolic	20 (13.16%)	25 (21.74%)	0.0708
Neoplastic	4 (2.63%)	-	0.1366
Viral	42 (27.63%)	31 (26.96%)	1.0000
Other	7 (4.61%)	1 (0.87%)	0.1433
d) Time post - tx (years)	4.94 ± 0.26	4.44 ± 0.30	0.2152
e) MELD score	21.25 ± 0.50	21.41 ± 0.58	0.8294
f) Immunosuppression (N)			
Steroids	3 (1.97%)	3 (2.61%)	1.0000
Tacrolimus	5 (3.29%)	4 (3.48%)	1.0000
Tacrolimus/Steroids	52 (34.21%)	29 (25.22%)	0.1396
Tacrolimus/Steroids/MMF	86 (56.58%)	70 (60.87%)	0.4524
Tacrolimus/MMF	2 (1.32%)	2 (1.74%)	1.0000
Tacrolimus/Sirolimus/Steroids	1 (0.66%)	2 (1.74%)	0.5782
Sirolimus/Steroids	1 (0.66%)	3 (2.61%)	0.3168
Sirolimus/Steroids/MMF	1 (0.66%)	1 (0.87%)	1.0000
Ciclosporina/Steroids/MMF	1 (0.66%)	-	1.0000
h) Biochemical enzymes (7 POD^a^)			
Alanine transaminase - ALT (U/L)	268.40 ± 15.16	213.63 ± 15.88	0.0132
Aspartate transaminase - AST (U/L)	91.05 ± 5.71	81.46 ± 9.55	0.3902
Alkaline phosphatase - ALP (U/L)	191.03 ± 10.51	152.29 ± 10.16	0.0085
Total bilirubin - TBIL (U/L)	2.76 ± 0.21	2.03 ± 0.23	0.0205
International normalized ratio (INR)	1.19 ± 0.01	1.12 ± 0.02	0.0033

MMF, mycophenolate mofetil; MELD, model for end-stage liver disease.

^a^ POD: postoperative days.

Regarding underlying disease etiology, alcoholic liver disease was the most frequent diagnosis among recipients in both age groups (25.66% in younger vs. 35.65% in elderly recipients; *p* = 0.0814), followed by viral hepatitis (27.63% vs. 26.96%; *p* = 1.000). Metabolic etiologies were more common in elderly recipients (21.74% vs. 13.16%; *p* = 0.0708), whereas autoimmune diseases were significantly more prevalent in younger recipients (21.05% vs. 5.22%; *p* = 0.0002). Neoplastic etiologies were reported solely in younger recipients (2.63%; *p* = 0.1366), and cryptogenic cirrhosis occurred with similar frequency in both groups (5.26% vs. 9.57%; *p* = 0.2298).

Immunosuppressive regimens were comparable between groups. The combination of tacrolimus, corticosteroids, and mycophenolate mofetil (MMF) was the most frequently prescribed regimen in both younger and elderly recipients (56.58% vs. 60.87%; *p* > 0.05). Younger recipients showed a higher tendency to receive tacrolimus plus corticosteroids without MMF (34.21% vs. 25.22%; *p* = 0.1396). Cyclosporine-based regimens were rare and used only in younger recipients. Monotherapy with corticosteroids or tacrolimus was infrequent but present in both age groups.

Postoperative day-7 biochemical parameters (ALT, ALP, TBIL and INR) were higher in younger recipients than in elderly recipients (*p* < 0.05). These values correspond to recipient-level postoperative measurements and are independent of donor liver age. This comparison is performed solely between young and elderly recipients, regardless of whether they received a young or an elderly graft.

### Perioperative ischemia parameters

Ischemia-related variables were considered procedural parameters and analyzed separately from donor and recipient characteristics. Warm ischemia time was comparable between younger and elderly donor grafts (41.65 ± 0.75 vs. 39.93 ± 1.62 min; *p* = 0.3444), while cold ischemia time showed only a modest tendency to be longer in younger-donor grafts (415.85 ± 7.20 vs. 398.56 ± 18.06 min; *p* = 0.3360).

### Reduced patient survival in recipients of elderly donor livers compared with recipients of younger donor livers

**FIGURE 2:**
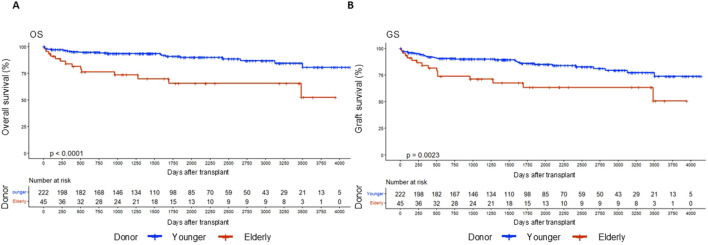
Patients who were transplanted using elderly livers presented reduced survival in contrast with the younger grafts. Comparison of OS **(A)** and GS **(B)** between recipients of livers 18 - 59 yo (group: Younger donor, n = 222) and recipients of older liver grafts ≥60 yo (group: Elderly donor, n = 45). Kaplan–Meier survival curves were used to analyze data.

To assess the impact of donor age on clinical outcomes following LTx, recipients were categorized into two groups based on donor age: the elderly donor group (donor ≥60 years; n = 45) and the younger donor group (donor 18–59 years; n = 222). Kaplan–Meier survival analyses showed significantly lower OS and GS in recipients of elderly donor livers compared with those receiving younger donor grafts (OS: *p* < 0.0001; GS: *p* = 0.0023; Figures 2A,B).

To identify independent predictors of OS and GS, we performed univariate and multivariable Cox regression analyses. Covariates included donor age, recipient age, donor gender, recipient gender, disease etiology (ALD, metabolic, autoimmune, or other), MELD score, year of transplantation, donor BMI, cold ischemia time, warm ischemia time, TBIL, and INR.

In the univariate analysis, donor age (HR 3.66, 95% CI 1.85–7.20; *p* = 0.0001) and TBIL (HR 4.98, 95% CI 1.51–16.4; *p* = 0.0081) were significantly associated with OS. For GS, donor age (HR 2.50, 95% CI 1.36–4.61; *p* = 0.0032), DRI (HR 1.95, 95% CI 1.09–3.46; *p* = 0.0224) and TBIL (HR 3.50, 95% CI 1.08–11.3; *p* = 0.0360) were significant.

**FIGURE 3:**
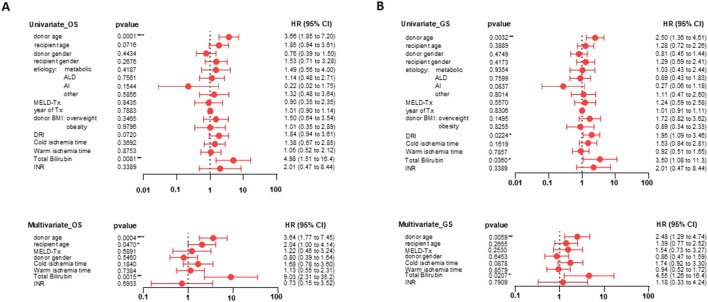
Univariate and multivariate Cox regression analyses of predictors of patient and GS. Forest plots show the results of univariate and multivariate analyses for OS **(A)** and GS **(B)**, presenting Hazard Ratios (HR) and their 95% Confidence Intervals (CI). For OS **(A)**, the univariate analysis showed significance for donor age (p = 0.0001) and TBIL (p = 0.0081), while the multivariate analysis identified donor age (p = 0.0004), recipient age (p = 0.0470), and TBIL (p = 0.0015) as independent predictors. For GS **(B)**, the univariate analysis showed significance for donor age (p = 0.0032), DRI (p = 0.0224), and TBIL (p = 0.0360), but only donor age (p = 0.0059) and TBIL (p = 0.0207) remained independent predictors in the multivariate analysis.

In the multivariable OS model, donor age (HR 3.64, 95% CI 1.77–7.45; *p* = 0.0004), recipient age (HR 2.04, 95% CI 1.00–4.14; *p* = 0.047), and TBIL (HR 9.03, 95% CI 2.31–35.2; *p* = 0.0015) remained independent predictors. In contrast, in the multivariable GS model, donor age (HR 2.48, 95% CI 1.29–4.74; *p* = 0.0059) and TBIL (HR 4.55, 95% CI 1.26–16.4; *p* = 0.0207) remained significant, whereas recipient age did not (HR 1.39, 95% CI 0.77–2.52; *p* = 0.2655) (Figures 3A,B).

To further clarify the role of recipient age, survival analyses were repeated within each donor-age stratum. There were no differences between younger and older recipients, whether they received elderly or a young donor liver (*p* > 0.05; Supplementary Figure S1A,BA).

Overall, these results indicate that elderly donor livers are associated with inferior OS and GS.

### Early allograft dysfunction and postoperative biochemical profiles in young vs. elderly donor livers

EAD was evaluated according to the Olthoff et al. (2010) criteria. EAD incidence remained low and did not differ between groups (elderly donors: 6.7% vs. younger donors: 2.3%; *p* > 0.05). The unadjusted odds ratio for EAD in elderly grafts was 3.10 (95% CI: 0.71–13.47). However, in the multivariable logistic model restricted to covariates with <30% missingness (cold ischemia time, MELD score, donor gender, and perioperative transfusions) donor age did not remain an independent predictor of EAD (*p* > 0.05) (Table 2).

**TABLE 2:** Early Allograft Dysfunction (EAD) and Renal Replacement Therapy (RRT) according to donor age.

Outcome	Young donors (n = 222)	Elderly donors (n = 45)	Effect size (elderly vs. young)
EAD	5 (2.3%)	3 (6.7%)	OR = 3.10; 95%CI 0.71–13.47
RRT ≤14d	0 (0%)	0 (0%)	Not estimable

Consistently, postoperative day-7 (POD7) biochemical parameters did not differ significantly between recipients of elderly and younger livers. ALT (247.08 ± 11.06 vs. 233.62 ± 28.75; *p* = 0.6512), AST (85.24 ± 4.99 vs. 95.18 ± 19.12; *p* = 0.4791), ALP (177.07 ± 8.29 vs. 161.44 ± 17.61; *p* = 0.4359), and TBIL (2.37 ± 0.16 vs. 2.77 ± 0.49; *p* = 0.2451) were comparable between groups. Taken together, these results indicate that both cohorts experienced a similar degree of early postoperative hepatocellular and cholestatic injury.

### Incidence of ACR during the first-year post-transplant

To evaluate the impact of donor age on alloimmune outcomes, the incidence of ACR during the first year after transplantation was assessed in the donor younger group (n = 222) and the donor elderly group (n = 45). A higher proportion of ACR was observed in the donor elderly group compared with the recipient younger group (42.86% vs. 28.24%), although this difference was not statistically significant (*p* > 0.05).

When stratified by recipient age, elderly recipients showed lower ACR rates than younger recipients (22.6% vs. 30.3%; *p* > 0.05), independently of graft age.

Specifically, ACR occurred in 19.78% of elderly recipients vs. 28.24% of younger recipients receiving younger donor livers, and in 33.33% vs. 42.86% of those receiving elderly donor livers (Table 3). These findings are consistent with the expected immunological attenuation associated with recipient aging.

**TABLE 3:** Rejection between donors groups vs*.* recipients groups.

Rejection		Donor	
		Younger	Elderly
Recipients	Younger	Rej 37 (28.24%)	Rej 9 (42.86%)
	Elderly	Rej 18 (19.78%)	Rej 8 (33.33%)

### Elevated pro-inflammatory cytokine levels in elderly liver grafts

**FIGURE 4:**
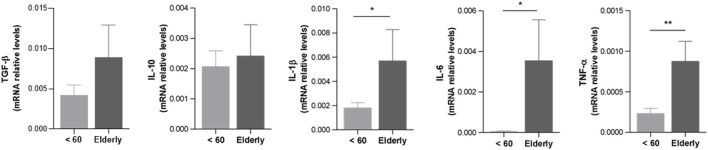
Levels of pro-inflammatory cytokines are elevated in patients with elderly liver compared to those with younger grafts. Pro-inflammatory (IL-1β, IL-6, and TNF-α) and immunoregulatory cytokines (TGF-β and IL-10) expression were evaluated by qRT-PCR in hepatic tissue obtained from younger and elderly grafts before their implantation into the recipient. Gene expression levels were determined by 2^−ΔCt^ method using GAPDH as normalizer gene. Statistics were calculated using an unpaired, two-tailed Mann-Whitney test depicted when p < 0.05 between groups. For p values: * indicates *p* < 0.05, ** indicates *p* < 0.01. Young donor group, n = 9; Elderly donor group, n = 5.

Cytokine expression levels were evaluated in liver tissue obtained from younger and elderly donor livers prior to implantation (Figure 4). Elderly donor livers showed significantly higher expression of the pro-inflammatory cytokines IL-1β, IL-6, and TNF-α (*p* < 0.05) compared with younger donor livers. In contrast, IL-10 expression was similar between groups, and TGF-β showed a non-significant upward trend in elderly grafts.

Overall, these findings indicate that elderly donor livers display a stronger basal pro-inflammatory cytokine profile than younger donor livers at the time of procurement, whereas regulatory cytokine expression remains largely unchanged.

### Accumulation of lipofuscin-positive cells and upregulation of cell cycle-related genes (p21 and CCND1) in the elderly group

**FIGURE 5:**
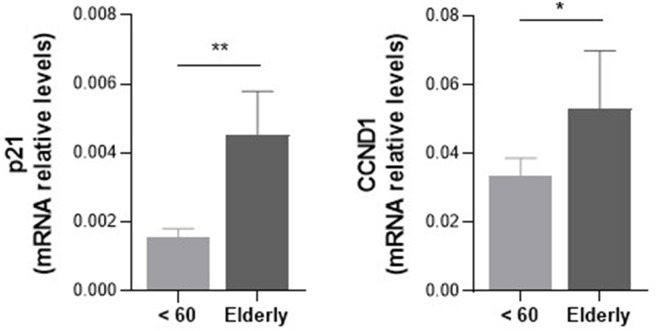
Higher levels of senescence markers in old livers compared to younger grafts. P21 and CCND1 expression were evaluated by qRT-PCR in hepatic tissue obtained from younger and elderly grafts before their implantation into the recipient. Gene expression levels were determined by 2^−ΔCt^ method using GAPDH as normalizer gene. Statistics were calculated using an unpaired, two-tailed Mann-Whitney test depicted when *p* < 0.05 between groups. For p values: ** indicates *p* < 0.01. Young donor group, n = 9; Elderly donor group, n = 5.

**FIGURE 6:**
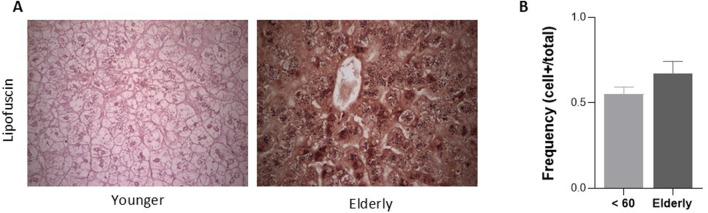
Lipofuscin-Positive Cells are more abundant in the elderly donor group compared to the younger grafts. **(A)** Representative photographs of lipofuscin staining in liver biopsies from young and elderly groups. **(B)** Lipofuscin-positive cells frequency in liver tissue, *p* < 0.05, Mann-Whitney test. Young donor group, n = 6; Elderly donor group, n = 6.

Senescence-associated markers were evaluated in liver tissue from younger and elderly donors (Figures 5, 6). Elderly donor livers demonstrated higher expression of the cell-cycle–related genes p21 and CCND1 compared with younger donor livers (*p* < 0.05; Figure 5). In addition, elderly donor livers showed a greater number of lipofuscin-positive cells, although this difference did not reach statistical significance (*p* > 0.05; Figure 6).

Overall, these findings indicate that elderly donor livers exhibit higher levels of senescence-associated molecular and histological markers at baseline.

## Discussion

The present study examined the impact of donor age on clinical outcomes in a single-center cohort of liver transplant recipients and integrated these findings with exploratory molecular and immunohistochemical analyses of donor liver tissue.

First, donor age ≥60 years was independently associated with reduced long-term overall and GS. This effect persisted after adjustment for donor-, recipient-, and transplant-related covariates, highlighting donor age as a robust determinant of late outcomes. The consistency of these results with prior multicenter and registry reports further reinforces the relevance of graft aging as a clinically meaningful dimension in LTx.

Second, the incidence of EAD did not differ between donor-age groups. In contrast, postoperative day-7 biochemical parameters are higher in recipients of younger livers. These biochemical differences cannot be attributed to donor-age effects and do not indicate an early functional disadvantage in elderly grafts.

Third, elderly grafts exhibited a distinct aging-associated molecular profile, with increased expression of pro-inflammatory cytokines and senescence-related markers, together with greater lipofuscin accumulation. This pattern is consistent with canonical features of hepatic aging—including inflammaging, mitochondrial vulnerability, reduced regenerative capacity, and impaired lysosomal clearance. Although exploratory, these findings provide biologically plausible mechanisms through which donor aging may compromise long-term graft resilience, despite the preservation of early postoperative function.

Finally, recipient age markedly modulated alloimmune responses, with older recipients experiencing lower rates of ACR. Together, these findings suggest that donor biological aging could exert a long-term influence on graft performance despite preserved early postoperative function, and that this effect may be shaped by the interplay between donor tissue biology and recipient immune competence.

The association between older donor age and reduced long-term graft and patient survival has been consistently demonstrated in large transplant registries and multicenter cohorts (Briceño et al., 2013; Xu et al., 2025; Feng et al., 2022; Jiang et al., 2020). Older livers accumulate structural and metabolic alterations including diminished regenerative capacity, mitochondrial dysfunction, impaired autophagy, and increased oxidative damage that may compromise long-term resilience (Lee et al., 2016; López-Otín et al., 2013). Our findings align with this literature, showing a clear decline in survival among recipients of grafts from donors ≥60 years.

Importantly, this association persisted after adjustment for available donor-, recipient-, and transplant-related variables. The uniformity of our cohort strengthens this interpretation: all donors were DBD, preservation relied exclusively on static cold storage using UW solution, and warm ischemia time was negligible. By reducing variability related to procurement and preservation, this study isolates donor age as a biologically relevant dimension rather than a surrogate for procedural heterogeneity.

Despite inferior long-term outcomes, elderly grafts in our cohort did not exhibit higher rates of EAD or early biochemical dysfunction. AST, ALT, bilirubin, INR, and PAL levels at 7 POD were comparable between groups, and the incidence of EAD did not differ. These findings contrast with the expected susceptibility of aged livers to ischemia–reperfusion injury but align with our observed EAD rates (6.7% vs. 2.3%, *p* > 0.05) and with recent evidence indicating that well-preserved DBD grafts from older donors may not experience disproportionate early injury (Feng et al., 2006; Han et al., 2021).

Several biological mechanisms may explain this apparent disconnect. Experimental models demonstrate that senescent hepatocytes exhibit reduced metabolic capacity and impaired mitochondrial stress responses, which together limit the release of intracellular enzymes even in the presence of significant cellular injury (Han et al., 2021; Schlegel et al., 2019). Consequently, elderly grafts may undergo a qualitatively distinct pattern of injury, more chronic, cumulative, and metabolically driven, rather than the acute, enzyme-releasing damage typically captured by early postoperative biochemical markers.

Recipient age strongly influenced the incidence of ACR. Older recipients exhibited substantially lower rejection rates, consistent with established features of immunosenescence, including diminished naïve T-cell pools, reduced proliferative capacity, and attenuated effector responses (Montecino-Rodriguez et al., 2013; Nikolich-Žugich, 2018). In contrast, donor age was not an independent predictor of ACR, and crude differences between age groups did not reach statistical significance. These data suggest that the pro-inflammatory milieu of aged grafts may be counterbalanced by the attenuated immune responsiveness of elderly recipients, resulting in a net neutral effect on rejection incidence.

Our findings also suggest that older grafts, although exhibiting a more pro-inflammatory baseline profile, do not provoke overtly higher rejection rates, possibly because this intrinsic inflammatory state is counterbalanced by the reduced immune competence of older recipients. In our cohort, elderly recipients were more likely to receive elderly grafts; Importantly, recipients of elderly grafts were themselves significantly older (a consequence of our nationally regulated allocation system, which does not permit center-level donor–recipient matching) and likely contributes to the attenuated rejection profile observed. However, this distribution reflects the national allocation system regulated by INCUCAI rather than center-driven matching. This represents an inherent limitation of our experimental design, as donor–recipient age pairings cannot be controlled by the transplant team and may introduce allocation-related bias that cannot be mitigated within this framework. Although donor–recipient age matching has been shown to reduce immnologic risk (Pagano et al., 2013; Busuttil and Tanaka, 2003) and improve utilization of older organs in other settings, in our context it arises from regulatory allocation rather than deliberate selection and must therefore be interpreted as a structural constraint of the study.

Another limitation is the retrospective, single-center design, which introduces potential selection and information bias. Moreover, several donor variables typically included in validated risk indices, such as detailed hemodynamic instability measures and comprehensive comorbidity scoring, were unavailable or inconsistently recorded, limiting the depth of donor-risk adjustment.

The transcriptional analyses showed that elderly grafts had increased expression of IL-1β, IL-6, and TNF-α, consistent with the “inflammaging” phenotype described in aging tissues (Franceschi et al., 2018; Prattichizzo et al., 2016). Although TGF-β is traditionally viewed as immunoregulatory, studies in aging organs indicate a shift toward senescence-associated and profibrotic signaling—promoting p21/p15 induction, matrix remodeling, and impaired regeneration (Tominaga and Suzuki, 2019; Mikuła-Pietrasik et al., 2022; Kiourtis et al., 2024), supporting that its mild upward trend here likely reflects age-related senescence. Together with the increased expression of p21 and CCND1 and greater lipofuscin accumulation, a marker of oxidative damage and impaired lysosomal clearance (Höhn and Grune, 2013; Porta, 2002), these findings support a senescence-enriched profile in elderly grafts and are consistent with their inferior long-term outcomes. Nevertheless, the molecular analyses were based on a small sample set and remain exploratory, requiring validation in larger cohorts with integrated omics and histopathology.

These findings provide biological plausibility for the survival disadvantage observed in elderly grafts. However, they must be interpreted with caution. The tissue analyses were performed on a limited subset of grafts with available samples, without longitudinal validation or functional assays. The data therefore represent hypothesis-generating evidence that aligns with established aging biology but does not establish mechanistic causality.

These results carry several clinical implications. First, older grafts can be safely used without increasing early postoperative complications under optimal procurement conditions. Second, in countries where donor–recipient matching is selectable, strategic age matching may improve long-term outcomes by avoiding the allocation of elderly grafts to younger, immunologically robust recipients, an approach that is not feasible in allocation systems like ours. Third, the molecular profile of elderly grafts highlights potential avenues for targeted interventions, including mitochondrial protection, senolytics, or tailored immunosuppression. Finally, as DCD use expands in our region, the interplay between donor age, warm ischemia, and perfusion technologies warrants further study.

In this uniform DBD cohort, donor age did not impair early graft function but was independently associated with reduced long-term graft and patient survival. Elderly grafts exhibited pro-inflammatory and senescence-associated profiles consistent with biological aging, providing a plausible mechanistic substrate for these outcomes. Recipient age strongly influenced alloimmune events, underscoring the complex interplay between donor tissue biology and host immune status. These findings highlight the importance of integrating biological aging markers into donor evaluation and allocation strategies and support the development of targeted approaches to optimize outcomes in LTx.

## Data Availability

The original contributions presented in the study are included in the article/Supplementary Material, further inquiries can be directed to the corresponding author/s.
