# Supplementary figures and images for: Age and liver transplantation: a key factor in clinical outcomes? single center study in Argentina
Source: Front Aging. 2025 Dec 11;6:1729048. doi: 10.3389/fragi.2025.1729048 (PMC12739379; doi:10.3389/fragi.2025.1729048)

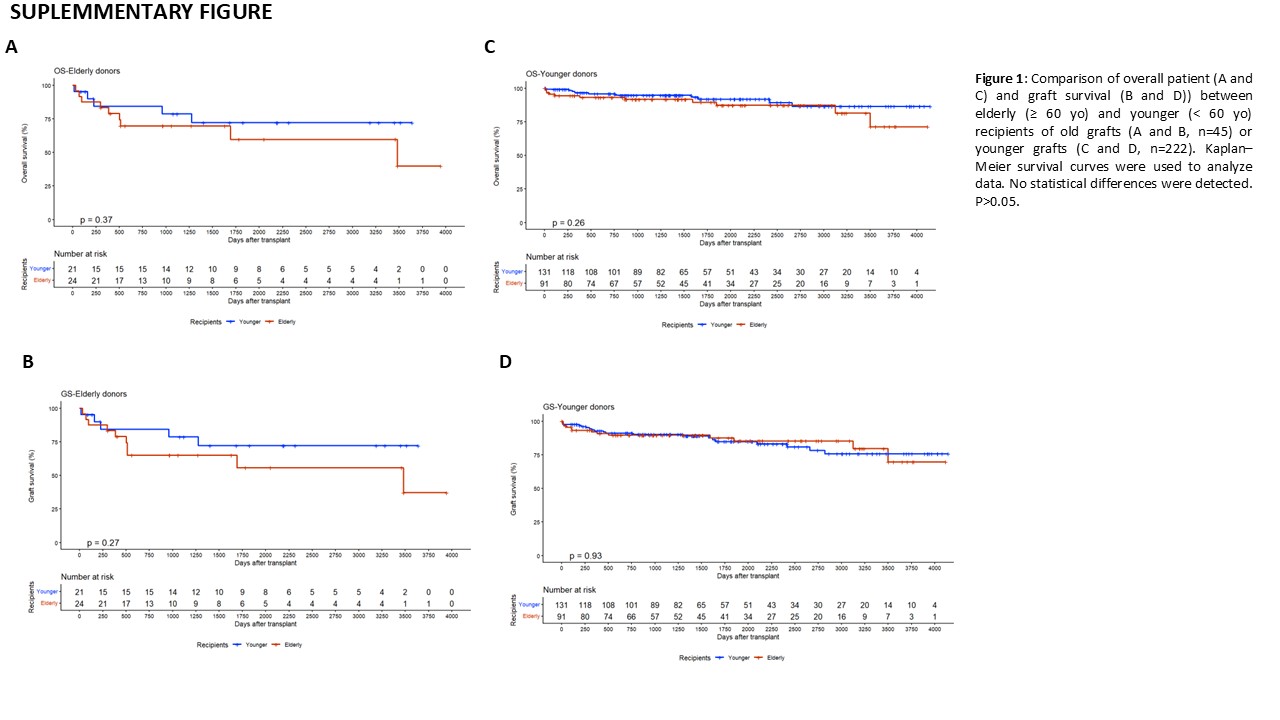

Supplement: Supplementary file 1 [file Image1.jpeg]
